# Transcriptional profiling of the model Archaeon *Halobacterium *sp. NRC-1: responses to changes in salinity and temperature

**DOI:** 10.1186/1746-1448-3-6

**Published:** 2007-07-25

**Authors:** James A Coker, Priya DasSarma, Jeffrey Kumar, Jochen A Müller, Shiladitya DasSarma

**Affiliations:** 1 University of Maryland Biotechnology Institute, Center of Marine Biotechnology, 701 East Pratt Street, Baltimore, MD 21202, USA; 2 Morgan State University, Department of Biology, 1700 East Cold Spring Lane, Baltimore, MD 21251, USA

## Abstract

**Background:**

The model halophile *Halobacterium *sp. NRC-1 was among the first Archaea to be completely sequenced and many post-genomic tools, including whole genome DNA microarrays are now being applied to its analysis. This extremophile displays tolerance to multiple stresses, including high salinity, extreme (non-mesophilic) temperatures, lack of oxygen, and ultraviolet and ionizing radiation.

**Results:**

In order to study the response of *Halobacterium *sp. NRC-1 to two common stressors, salinity and temperature, we used whole genome DNA microarrays to assay for changes in gene expression under differential growth conditions. Cultures grown aerobically in rich medium at 42°C were compared to cultures grown at elevated or reduced temperature and high or low salinity. The results obtained were analyzed using a custom database and microarray analysis tools. Growth under salt stress conditions resulted in the modulation of genes coding for many ion transporters, including potassium, phosphate, and iron transporters, as well as some peptide transporters and stress proteins. Growth at cold temperature altered the expression of genes involved in lipid metabolism, buoyant gas vesicles, and cold shock proteins. Heat shock showed induction of several known chaperone genes. The results showed that *Halobacterium *sp. NRC-1 cells are highly responsive to environmental changes at the level of gene expression.

**Conclusion:**

Transcriptional profiling showed that *Halobacterium *sp. NRC-1 is highly responsive to its environment and provided insights into some of the specific responses at the level of gene expression. Responses to changes in salt conditions appear to be designed to minimize the loss of essential ionic species and abate possible toxic effects of others, while exposure to temperature extremes elicit responses to promote protein folding and limit factors responsible for growth inhibition. This work lays the foundation for further bioinformatic and genetic studies which will lead to a more comprehensive understanding of the biology of a model halophilic Archaeon.

## Background

Halophilic archaea (haloarchaea) flourish in extremely saline environments and are exceptionally tolerant of many environmental stresses [1,2]. Among haloarchaea, several closely related *Halobacterium *species are the best-studied, display the greatest halophilicity, and are widely distributed in nature. They are typified by the well-studied model organism, *Halobacterium *sp. NRC-1, which grows fastest aerobically in amino acid-rich environments at moderate temperatures and nearly saturated brine [3]. This strain has the ability to survive and grow phototrophically using the light driven proton pumping activity of bacteriorhodopsin in its purple membrane and anaerobically via substrate level phosphorylation using arginine and by respiration using dimethyl sulfoxide (DMSO) and trimethylamine *N*-oxide (TMAO) [4,5]. It can also survive at temperatures spanning the range from 10 to 56°C and NaCl concentrations from 2.5 to 5.3 M (saturation). *Halobacterium *sp. NRC-1 is highly tolerant of both ultraviolet light and ionizing radiation, the latter of which may be related to its relatively high desiccation resistance [6-9]. The remarkable tolerance of *Halobacterium *sp. NRC-1 to multiple extremes distinguishes this organism among extremophiles and Archaea [1,2].

The *Halobacterium *NRC-1 genome sequence was completed in 2000 and found to be 2.57 Mb in size [10-12], and is composed of three circular replicons: a large chromosome (2.0 Mb) and two minichromosomes, pNRC100 (191 kb) and pNRC200 (365 kb). Analysis of the genome sequence identified 2,682 likely genes (including 52 RNA genes), of which 1,658 coded for proteins with significant matches to the database. Of the matches, 591 were to conserved hypothetical proteins, and 1067 were to proteins with known or predicted functions. Interestingly, about 40 genes on pNRC100 and pNRC200 coded for proteins likely to be essential or important for cell viability, indicating that these replicons function as minichromosomes. Bioinformatic analysis identified 149 likely regulators and multiple general transcription factors, including six TBPs and seven TFBs, which were hypothesized to regulate gene expression in response to environmental changes [13].

After complete sequencing of the *Halobacterium *sp. NRC-1 genome, key post-genomic methods were developed, including a facile gene knockout system for reverse genetic analysis [14,15], and a whole genome DNA microarray for transcriptomic analysis [5,13]. We employed the DNA microarray system developed by Agilent Corporation using inkjet technology for in situ synthesis of oligonucleotides directly on glass slides [16]. This system provides high specificity by use of 60-mer oligonucleotide probes and high data quality due to limited technical noise. In the present work, we have successfully used a platform containing 2 × 8,455 features per slide representing duplicate microarrays with 2474 (97%) open reading frames (ORFs). Up to four unique probes were designed per ORF with a mean T_*m*_of 81°C and a T_*m *_range of 3°C. The microarray performance was tested through linearity of response and statistical significance using both biological and technical replicates [5].

We conducted three previous whole genome transcriptomic studies to examine the response of *Halobacterium *sp. NRC-1 to extreme conditions using DNA microarrays [5-7]. In the first study, we investigated cell growth by anaerobic respiration on either DMSO or TMAO as the sole terminal electron acceptor, and found the requirement of the *dms*REABCD operon for growth under anaerobic respiration. Whole genome DNA microarray analysis showed that the *dms *operon is highly induced when cells are grown anaerobically with TMAO and comparison of *dms*R^+ ^and Δ*dms*R strains showed that the induction of the *dms*EABCD operon is dependent on a functional *dms*R gene, consistent with its action as a transcriptional activator. Expression of the purple membrane protein bacterio-opsin (*bop*) gene as well as genes specifying buoyancy conferring gas vesicles were also induced under limiting oxygen conditions, indicating that cells respond by moving to more aerobic and illuminated zones where the alternate physiological capabilities may be utilized.

We also studied the response of *Halobacterium *sp. NRC-1 to high levels of UV radiation damage, an environmental stress that results from solar radiation present in its environment [6]. Cells were irradiated with 30–70 J/m^2 ^UV-C, and transcriptional profiling showed the most strongly up-regulated gene was *rad*A1, the archaeal homolog of *rad*51 in eukaryotes and *rec*A in bacteria. Additional genes involved in homologous recombination, such as *arj*1 (*rec*J-like exonuclease), *dbp *(eukaryote-like DNA binding protein of the superfamily I DNA and RNA helicases), and *rfa*3 (replication protein A complex), as well as *nrd*J, (cobalamin-dependent ribonucleotide reductase involved in DNA metabolism), were also significantly induced. Neither prokaryotic nor eukaryotic excision repair gene homologs were induced and there was no evidence of an SOS-like response. These results showed that homologous recombination plays an important role in the cellular response of *Halobacterium *sp. NRC-1 to UV damage.

In our most recently published study [7], we generated and examined mutants of *Halobacterium *sp. NRC-1 that are resistant to high energy ionizing radiation. Two independently-obtained mutants displaying LD_50_>11 kGy, which is higher than that of the extremely radiation-resistant bacterium *Deinococcus radiodurans*, were found to up-regulate an operon comprised of two single-stranded DNA binding protein (RPA) genes, *rfa*3, *rfa*8, and a third gene, *ral*, of unknown function. These results suggested that RPA facilitates DNA repair machinery and/or protects repair intermediates to maximize the ionizing radiation-resistance of this archaeon.

In the current report, we used whole genome DNA microarrays for *Halobacterium *sp. NRC-1 to assay the changes in gene expression in response to several common environmental conditions, high and low salinity and temperature. These data serve as a significant resource to expand our understanding of the physiological and transcriptional responses of *Halobacterium *sp. NRC-1 to the wide range of environmental stresses to which it is exposed.

## Results and discussion

### Low and high salinity

*Halobacterium *sp. NRC-1 flourishes in environments that are not only hypersaline (4.3 M NaCl optimum) but also highly variable in total salinity (2.5–5.3 M NaCl) as a result of common evaporatic and dilution processes [1]. Therefore, the cells must cope with high as well as dynamic concentrations of dissolved salts. The intracellular concentration of KCl is roughly equal (concentrations up to 5 M have been reported) to the external NaCl concentrations, and is used as the major compatible solute [17]. Genome-wide, predicted proteins have a median pI of 4.9, with a concentration of negative charges on their surface, characteristics which permit effective competition for hydration and allow function in a cytoplasm with low water activity [3,18]. High salt concentrations are also known to convert some DNA sequences, e.g. alternating (CG)-repeats, from the right-handed B to the left-handed Z form, facilitated by negative superhelical stress [19,20]. Organic compatible solutes commonly utilized in the response of bacterial and eukaryotic halophiles to osmotic stress, have also been reported in *Halobacterium *sp. NRC-1 [21,22].

The annotated genome of *Halobacterium *sp. NRC-1 provided an inventory of likely genes involved in maintaining the intracellular ionic conditions suitable for growth [11,12]. Genes coding for multiple active K^+ ^transporters were found, including *kdp*ABC, an ATP-driven K^+ ^uptake system, and *trk*AH, low-affinity K^+ ^transporters driven by the membrane potential. Genes coding for an active Na^+ ^efflux system likely mediated by NhaC proteins were also present, corresponding to the unidirectional Na^+^-H^+ ^antiporter activity described previously [23]. Interestingly, several of these genes, including those coding for *kdp*ABC, *trk*A (three copies), and *nha*C (one copy) were found on the extrachromosomal pNRC200 replicon. However, a comprehensive study of the adaptation of *Halobacterium *sp. NRC-1 to various salinities was not previously reported.

**Figure 1:**
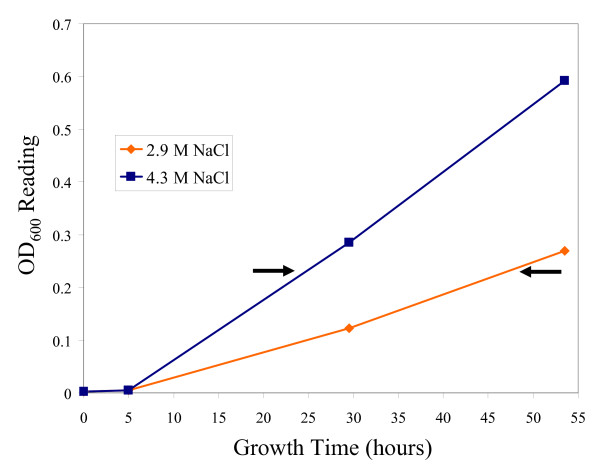
Growth curves of *Halobacterium *sp. NRC-1 cultures at low salt conditions (2.9 M NaCl) and standard conditions (4.3 M NaCl). Arrows indicate the point at which cultures were harvested for microarray analysis.

**Figure 2:**
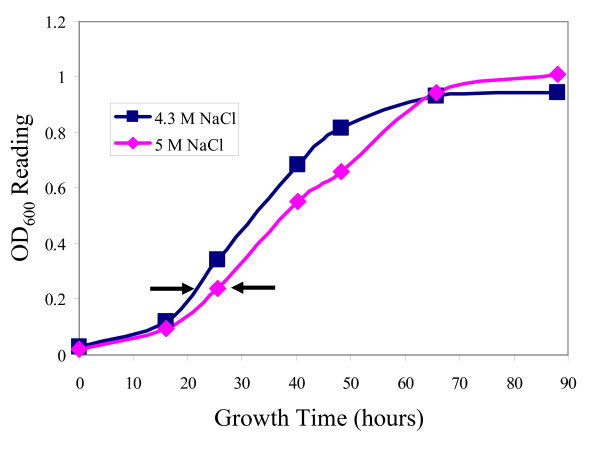
Growth curves of *Halobacterium *sp. NRC-1 cultures at high salt conditions (5.0 M NaCl) and standard conditions (4.3 M NaCl). Arrows indicate the point at which cultures were harvested for microarray analysis.

**Figure 3:**
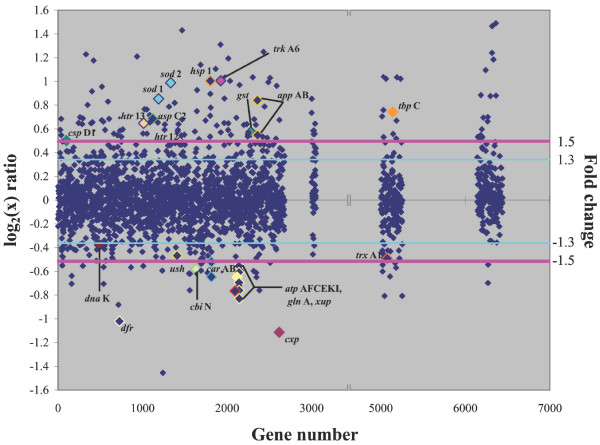
Scatter plot comparing DNA microarrays hybridized with cDNA from *Halobacterium *sp. NRC-1 cultures grown in low (2.9 M) and standard (4.3 M) NaCl conditions. The log_2 _value of the *Cy*5/*Cy*3 ratio for each gene (abscissa) is plotted versus the gene number (ordinate). Gene numbers correspond to the chromosomal genes (1–2679), RNA genes (3000 to 3051), pNRC100 genes (5000 to 5256), and pNRC200 genes (6000 to 6487). Pink line represents a fold change value of 1.5 and the light blue line represents a fold change value of 1.3. Colors have been added for emphasis.

**Figure 4:**
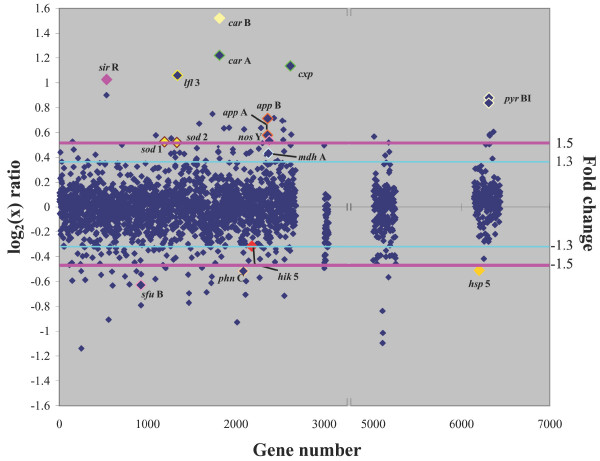
Scatter plot comparing DNA microarrays hybridized with cDNA from *Halobacterium *sp. NRC-1 cultures grown in high (5 M) and standard (4.3 M) NaCl. The log_2 _value of the *Cy*5/*Cy*3 ratio for each gene (abscissa) is plotted versus the gene number (ordinate). Gene numbers correspond to the chromosomal genes (1–2679), RNA genes (3000 to 3051), pNRC100 genes (5000 to 5256), and pNRC200 genes (6000 to 6487). Pink line represents a fold change value of 1.5 and the light blue line represents a fold change value of 1.3. Colors have been added for emphasis.

In order to determine which genes were most responsive to high and low salinity, *Halobacterium *sp. NRC-1 cultures were grown at three salinities, low (2.9 M NaCl), optimal (4.3 M NaCl), and high (5.0 M NaCl), in rich media at 42°C (Figs. 1 &2). The high and low salinity conditions were selected to ensure that salts did not precipitate and cells did not lyse during culture, respectively. Growth at low salinity compared to optimal salinity displayed 143 up-regulated genes and 53 down-regulated genes by 1.5-fold or greater (Fig. 3). Growth at high salinity, where the NaCl concentration (5 M) was only slightly higher than the optimal salinity (4.3 M) displayed 32 up-regulated genes and 29 down-regulated genes by 1.5-fold or greater (Fig. 4).

Transcription of several potassium and sodium ion transporter genes was affected by salt conditions in NRC-1, mainly by low salt [24,25]. Among the putative potassium transporters, the *trk*A6 gene, was significantly up-regulated (2.0-fold) under low salt conditions (Table 1). This gene product may compensate for the loss of K^+ ^under low salt conditions, via membrane leakiness, by uptake of more potassium ions. Among sodium transporters, only one (*nha*C3, coded by pNRC200) gene was slightly down-regulated (1.3-fold) under low salt, while the others were unchanged. The lack of a more vigorous induction of the sodium-proton antiporter suggests that increases of Na^+ ^concentration in cells may be tolerated during osmotic stress, possibly substituting for K^+ ^intracellularly.

**Table 1:** Selected genes significantly altered under low salinity growth

Gene ID	Gene name	Former name	COG	Predicted function	Fold change values	log_2_(x) ratio	Standard deviation of log_2_(x) ratio
101	cspD1		1278	cold shock protein	1.442	0.502	0.275
491	dnaK		443	heat shock protein	-1.307	-0.376	0.172
727	dfr		534	DinF-related damage-inducible protein	-2.079	-1.019	0.316
1013	htr13		840	Htr13 transducer	1.656	0.649	0.482
1121	aspC2		436	aspartate aminotransferase	1.694	0.688	0.453
1190	sod1		605	superoxide dismutase	1.826	0.852	0.221
1332	sod2		605	superoxide dismutase	2.023	0.988	0.290
1408	ush		737	UDP-sugar hydrolase	-1.393	-0.468	0.165
1442	htr12		840	Htr12 transducer	1.406	0.490	0.058
1632	cbiQ		619	cobalt transport protein	-1.265	-0.324	0.214
1634	cbiN		1930	cobalt transport protein	-1.553	-0.580	0.400
1691	rpl23p		89	50S ribosomal protein L23P	-1.454	-0.533	0.138
1692	rpl2p		90	50S ribosomal protein L2P	-1.515	-0.594	0.128
1695	rpl22p		91	50S ribosomal protein L22P	-1.447	-0.523	0.173
1698	rpl29p		255	50S ribosomal protein L29P	-1.460	-0.535	0.176
1801	hsp1		71	small heat shock protein	2.248	1.006	0.679
1814	carB		458	carbamoyl-phosphate synthase large subunit	-1.590	-0.646	0.261
1815	carA		505	carbamoyl-phosphate synthase small subunit	-1.452	-0.520	0.225
1924	trkA6		569	TRK potassium uptake system protein	2.040	1.006	0.251
2093	glnA		174	glutamine synthatase	-1.719	-0.766	0.212
2116	xup		2252	xanthine/uracil permease family protein	-1.603	-0.647	0.307
2139	atpA		1155	H^+^-transporting ATP synthase subunit A	-1.482	-0.555	0.189
2140	atpF		1436	H^+^-transporting ATP synthase subunit F	-1.536	-0.608	0.177
2141	atpC		1527	H^+^-transporting ATP synthase subunit C	-1.637	-0.694	0.221
2142	atpE		1390	H^+^-transporting ATP synthase subunit E	-1.706	-0.761	0.173
2143	atpK		636	H^+^-transporting ATP synthase subunit K	-1.658	-0.719	0.173
2144	atpI		1269	H^+^-transporting ATP synthase subunit I	-1.816	-0.830	0.295
2281	gst		435	glutathione S-transferase	1.508	0.573	0.235
2344	dppD1	oppD2	444	oligopeptide ABC transporter	-1.562	-0.611	0.301
2346	dppC1	dppC2	1173	dipeptide ABC transporter permease	-1.436	-0.488	0.307
2347	dppB1	dppB1	601	dipeptide ABC transporter permease	-1.581	-0.649	0.182
2358	appA		747	oligopeptide binding protein	1.893	0.841	0.485
2359	appB		601	oligopeptide ABC permease	1.506	0.548	0.350
2616	cxp		2317	Carboxypeptidase	-2.194	-1.113	0.247
5076	trxA1			Thioredoxin	-1.424	-0.491	0.235
5142	tbpC		2101	transcription initiation factor IID	1.748	0.743	0.430
6313	nhaC3		1757	Na^+^/H^+ ^antiporter	-1.280	-0.348	0.146

Expression of many other transporter genes was altered by growth in non-optimal salinities. The *sfu*B iron transporter-like permease protein was down-regulated (1.6-fold) under high salt (Table 2), and the *phn*C gene, a component of the phosphate/phosphonate transport system was down-regulated by 1.4-fold in high salt, suggesting that *Halobacterium *sp. NRC-1 responds to osmotic stress by reducing uptake of these species, which may potentially be toxic [26]. The *nos*Y gene, coding for an apparent ABC transporter component for nitrite/nitrate, was up-regulated under high salt (1.5-fold), although usage of nitrite or nitrate for respiration or as a nitrogen source has not been detected for NRC-1 [13]. Members of the two similarly organized gene clusters containing oligopeptide/dipeptide/Ni^2+ ^transporter genes (*app*ABCDF and *dpp*ABCDF, see Table 1), were responsive to salinity changes. Two genes in the *app *cluster were up-regulated between 1.5- and 1.9-fold under both high and low salt, while some genes in the *dpp *cluster were down-regulated 1.4- to 1.6-fold under low salt. In both cases, the permease genes, C and/or B, were more affected than the genes coding for the periplasmic and ATPase components. The increase in expression of these genes is consistent with an attempt by cells to minimize the loss of these ionic (or zwitterionic amino acids) species, whereas down-regulation may be related to growth rate or possible toxic effects. Interestingly, the *pro*X and *htr*5 genes, which are proposed to code for compatible solute (possibly trimethylammonium) binding and transporter proteins (*cos*B and *cos*T, respectively, in the closely related *Halobacterium salinarium *[27]) were unchanged in high or low salt concentrations in NRC-1.

**Table 2:** Selected genes significantly altered under high salinity growth

Gene ID	Gene name	Former name	COG	Predicted function	Fold change values	log_2_(x) ratio	Standard deviation of log_2_(x) ratio
536	sirR		1321	transcription repressor	2.048	1.026	0.146
923	sfuB		1178	iron transporter-like protein	-1.598	-0.628	0.384
1190	sod1		605	superoxide dismutase	1.441	0.525	0.073
1332	sod2		605	superoxide dismutase	1.435	0.521	0.045
1339	lfl3		318	long-chain fatty-acid-CoA ligase	2.139	1.060	0.325
1814	carB		458	carbamoyl-phosphate synthase large subunit	2.915	1.521	0.261
1815	carA		505	carbamoyl-phosphate synthase small subunit	2.532	1.222	0.595
2085	phnC		3638	phosphonate transport ATP-binding	-1.432	-0.516	0.076
2180	hik5	vng2180	642	sensory histidine protein kinase (HisKA domain)	-1.246	-0.315	0.072
2358	appA		747	oligopeptide binding protein	1.525	0.579	0.290
2359	appB		601	oligopeptide ABC permease	1.687	0.713	0.339
2367	mdhA		39	L-malate dehydrogenase	1.351	0.432	0.081
2377	nosY		1277	nitrite/nitrate ABC transporter	1.453	0.534	0.124
2616	cxp		2317	carboxypeptidase	2.210	1.137	0.143
6201	hsp5		71	heat shock protein	-1.427	-0.510	0.093
6309	pyrB		540	aspartate carbamoyltransferase catalytic subunit	1.803	0.838	0.190
6311	pyrI		1781	aspartate carbamoyltransferase regulatory chain	1.847	0.879	0.128

Protein kinases are believed to be involved in the salt stress response cascade in eukaryotic cells [28] as well as some prokaryotic species [29]. Among genes possibly involved in signaling in *Halobacterium *sp. NRC-1, the only signal transduction histidine kinase gene out of 13 in the genome that showed any significant change was *hik*5, for which expression under the high salt condition was slightly lowered (1.3-fold). In the large halotransducer (*htr*) family in NRC-1 only two of 17 *htr *genes, *htr*12 and *htr*13, were up-regulated under low salt (1.4- to 1.7-fold) [30]. Knowledge of the true functions of these families of genes and their precise roles in signal transduction in *Halobacterium *sp. NRC-1 is quite limited at present.

Several transcription factors and regulators were significantly changed under salt stress conditions. Among these, one basal transcription factor gene out of 13 in NRC-1 [12], *tbp*C (coded by pNRC200), was up-regulated 1.7-fold under low salt conditions. Also, the *sir*R gene, a putative transcriptional repressor, was significantly up-regulated under high salt (2.0-fold).

Interestingly, three stress genes were inducible under high and low salt conditions. Both *sod *genes, which encode superoxide dismutase, were salt-responsive. The *sod*2 transcript was up-regulated 1.4-fold under high salt and 2.0-fold under low salt, and *sod*1 was up-regulated 1.4-fold under high salt and 1.8-fold under low salt. Superoxide dismutase is responsible for limiting damage from reactive oxygen species, which are induced by extreme conditions, such as oxidative stress and excess irradiance [31] in many species. The *gst *gene, coding for glutathione S-transferase, another detoxification enzyme involved in cell protection from reactive oxygen species [32], was up-regulated (1.5-fold) under low salt conditions. These findings suggest that the removal of reactive oxygen species is of increased importance in osmotically stressed haloarchaea.

An earlier study [33] suggested that the small heat shock protein family coded by *hsp *genes may be involved in salt stress responses in the related haloarchaeon, *Haloferax volcanii*. We observed that two *hsp *genes in *Halobacterium *sp. NRC-1 were affected by salinity changes: *hsp*1 was up-regulated under low salt conditions (2.3-fold) and *hsp*5, coded by pNRC200, was down-regulated 1.4-fold under high salt conditions (as well as in the cold, see below). Two predicted stress genes, the cold shock gene *csp*D1 (1.4-fold up-regulated), and the heat shock gene *dna*K (1.3-fold down-regulated), were regulated under low salt conditions, which suggests a wider role than temperature adaptation for these chaperones. Interestingly, a carboxypeptidase gene, *cxp*, which may function in protein turnover, was highly down-regulated in low salt (2.2-fold), and equally up-regulated in high salt.

Expression of a number of genes involved in cellular metabolism was significantly altered by salinity changes. Two highly regulated genes, *car*A and *car*B, coding for both subunits of carbamoyl-phosphate synthase, were up-regulated between 2.5- and 2.9-fold under high salt conditions and down-regulated 1.5- to 1.6-fold under low salt conditions. Any relation of these results to similar observations in *Xenopus *is unclear [34]. One sugar kinase, product of the *ush *gene, was down-regulated (1.4-fold) at low salinity, and the malate dehydrogenase gene, *mdh*A, was up-regulated (1.4-fold) at high salt in NRC-1. The long-chain fatty acid-CoA ligase, coded by *lfl*3, was up-regulated 2.1-fold in high salt. However, we did not observe a similar change in other genes involved in lipid metabolism [35]. The glutamine synthetase gene, *gln*A, was down-regulated (1.7-fold) under low salt conditions. The thioredoxin gene, *trx*A1, which is present on the inverted repeats of the pNRC replicons, was down-regulated under low salt conditions (1.4-fold). One of two aspartate aminotransferase genes, *asp*C2, was up-regulated under low salt (1.7-fold), and the two subunits of aspartate carbamoyltransferase, coded by *pyr*B and *pyr*I (both coded by pNRC200), were up-regulated under high salt (1.8-fold). A xanthine/uracil permease family protein, coded by *xup*, was down-regulated by 1.6-fold under low salt conditions, and the Na^+^-driven multidrug efflux pump, coded by the *dfr *gene, was down-regulated by 2.1-fold also under low salt. The *cbi*NQ genes, coding for part of the Co^2+ ^transport system, were down-regulated 1.3- to 1.6-fold in low salt; however, this result may reflect the lower growth rate. Additional likely growth-rate dependent genes altered under low salt, included genes coding for the large ribosomal proteins, *rpl*2p, *rpl*22p, *rpl*23p, *rpl*29p (about 1.5-fold down-regulated) and the H^+^-transporting ATP synthase subunits, *atp*IKECFA (1.5 to 1.8-fold down-regulated). It is possible that the changes in gene expression of many of these salt-responsive metabolic genes in NRC-1 may be indirect, most likely as a result of growth rate changes.

### Growth in the cold

Optimum growth for *Halobacterium *sp. NRC-1 occurs at 42°C, but measurable growth is observed down to a temperature of 15°C, with very slow growth at temperatures as low as 10°C [11,36]. The growth temperature optimum and minimum for NRC-1 are typical of most haloarchaea, but significantly higher than for some cold-adapted species, e.g. *Halorubrum lacusprofundi*, where the temperature minimum for growth has been reported down to -2°C [36,37].

**Figure 5:**
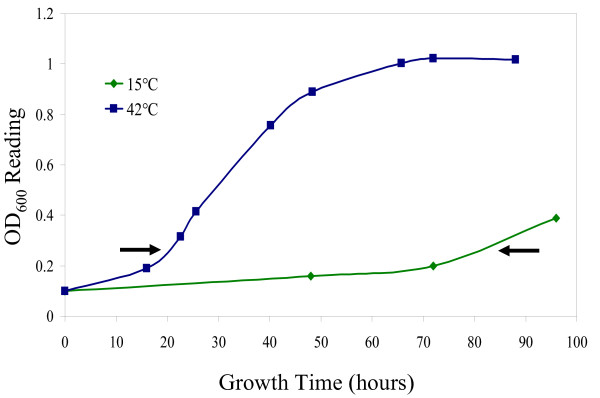
Growth curves of *Halobacterium *sp. NRC-1 cultures grown in cold (15°C) and standard (42°C) temperatures. Arrows indicate the point where cultures were harvested for microarray analysis.

**Figure 6:**
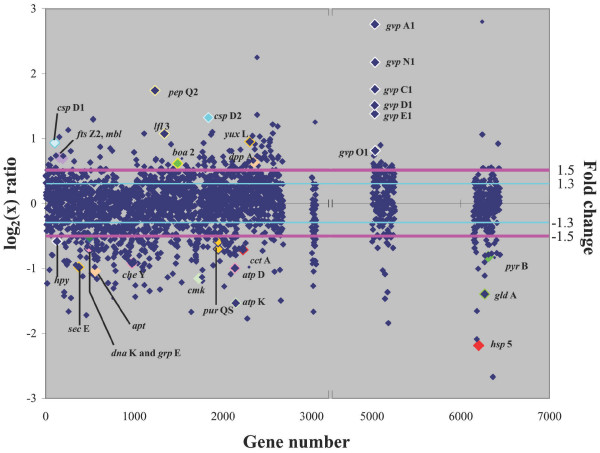
Scatter plot comparing DNA microarrays hybridized with cDNA from *Halobacterium *sp. NRC-1 cultures grown in cold (15°C) and standard (42°C) temperatures. The log_2 _value of the *Cy*5/*Cy*3 ratio for each gene (abscissa) is plotted versus the gene number (ordinate). Gene numbers correspond to the chromosomal genes (1–2679), RNA genes (3000 to 3051), pNRC100 genes (5000 to 5256), and pNRC200 genes (6000 to 6487). Pink line represents a fold change value of 1.5 and the light blue line represents a fold change value of 1.3. Colors have been added for emphasis.

In order to determine which genes in *Halobacterium *sp. NRC-1 are responsive to reduced temperatures, cultures were grown at low (15°C) and optimal (42°C) temperatures (Fig. 5). Growth at 15°C resulted in up-regulation of 151 genes and down-regulation of 287 genes by 1.5-fold or greater (Fig. 6). Interestingly, 29% of the up-regulated and 37% of the down-regulated genes are of unknown function, suggesting the involvement of novel genes in adaptation of *Halobacterium *sp. NRC-1 to cold temperature.

An interesting class of responsive genes was those involved in membrane lipid metabolism. The gene coding for the first step in synthesis of polar lipids, *sn*-1-glycerol phosphate dehydrogenase (*gld*A), which is coded by pNRC200, was down regulated 2.7-fold (Table 3), suggesting that polar lipid biosynthesis is altered in *Halobacterium *sp. NRC-1 cells growing at low temperature. There is also an up-regulation of genes encoding dehydrogenases, such as *acd*4 (1.5-fold) coding for acyl-CoA dehydrogenase, and genes coding for increased turnover of polar lipids, e.g. *lfl*3 (2.2-fold), coding for a long-chain fatty acid-CoA ligase, and *aca *(1.4-fold), coding an acetoacetyl-CoA thiolase. These results are consistent with *Halobacterium *sp. NRC-1 altering the composition of its lipids in the cold, as has been previously reported in other microorganisms [38-42].

**Table 3:** Selected genes significantly altered under low temperature growth

Gene ID	Gene name	Former name	COG	Predicted function	Fold change values	log_2_(x) ratio	Standard deviation of log_2_(x) ratio
101	cspD1		1278	cold shock protein	2.307	0.935	0.912
134	hpyA		2036	archaeal histone	-1.525	-0.584	0.269
153	mbl	vng153		mreB-like protein	1.685	0.708	0.365
192	ftsZ2		206	cell division protein	1.635	0.696	0.203
254	tfbG		1405	transcription initiation factor IIB	2.259	1.132	0.356
375	secE		2443	protein translocase	-2.004	-0.982	0.244
491	dnaK		443	heat shock protein	-1.654	-0.695	0.303
494	grpE		576	heat shock protein	-1.477	-0.522	0.353
559	apt		503	adenine phosphoribosyltransferase	-2.143	-1.041	0.410
679	acd4		1960	acyl-CoA dehydrogenase	1.516	0.531	0.477
683	fbaA		1830	possible 1,6-fructose biphosphate aldolase	-1.606	-0.673	0.171
823	gspE2		0630	type II secretion system protein	-1.624	-0.623	0.472
887	gyrB		187	DNA gyrase subunit B	-1.422	-0.458	0.397
905	pmu2		1109	phosphomannomutase	-1.800	-0.833	0.206
974	cheY		784	chemotaxis protein	-1.956	-0.923	0.349
1233	pepQ2		6	X-pro aminopeptidase homolog	3.430	1.741	0.330
1339	lfl3		318	long-chain fatty-acid-CoA ligase	2.174	1.075	0.366
1488	boa2		3413	bacterio-opsin activator-like protein	1.551	0.612	0.231
1690	rpl4e		0088	50S ribosomal protein L4E	-1.685	-0.675	0.474
1703	rps4e		1471	30S ribosomal protein S4E	-1.598	-0.599	0.477
1727	cmk		1102	cytidydylate kinase	-2.254	-1.154	0.231
1836	cspD2		1278	cold shock protein	3.112	1.322	0.990
1944	purS	vng1944	1828	phosphoribosylformylglycinamidine synthase, PurS component	-1.523	-0.606	0.032
1945	purQ	purL2	47	phosphoribosylformylglycinamide synthase I, glutamine amidotransferase domain	-1.509	-0.590	0.108
2063	aca		183	probable acetyl-CoA acetyltransferase	1.421	0.483	0.263
2135	atpD		1394	H^+^-transporting ATP synthase subunit D	-2.012	-0.998	0.173
2138	atpB		1156	H^+^-transporting ATP synthase subunit B	-1.381	-0.449	0.221
2142	atpE		1390	H^+^-transporting ATP synthase subunit E	-1.716	-0.728	0.389
2143	atpK		636	H^+^-transporting ATP synthase subunit K	-2.926	-1.535	0.203
2144	atpI		1269	H^+^-transporting ATP synthase subunit I	-1.802	-0.798	0.388
2226	cctA		459	Thermosome subunit alpha	-1.735	-0.713	0.489
2302	yuxL		1506	acylaminoacyl-peptidase	2.096	0.951	0.601
2349	dppA		747	dipeptide ABC transporter dipeptide-binding	1.609	0.634	0.486
5028	gvpE1			GvpE protein	3.097	1.381	0.883
5029	gvpD1			GvpD protein	3.396	1.513	0.875
5030	gvpA1			GvpA protein	8.018	2.761	0.860
5032	gvpC1			GvpC protein	3.985	1.761	0.844
5033	gvpN1			GvpN protein	5.161	2.174	0.764
5034	gvpO1			GvpO protein	1.824	0.814	0.395
6201	hsp5		71	heat shock protein	-4.784	-2.187	0.446
6270	gldA		371	sn-glycerol-1-phosphate dehydrogenase	-2.676	-1.396	0.266
6309	pyrB		540	aspartate carbamoyltransferase catalytic subunit	-1.767	-0.817	0.121
6311	pyrI		1781	aspartate carbamoyltransferase regulatory chain	-1.835	-0.855	0.244

The genomes of several haloarchaea have shown the presence of multiple cold shock genes, the products of which may bind to single-stranded DNA and RNA, functioning as RNA chaperones, and facilitating the initiation of translation under low temperatures [36,43,44]. As expected, expression of both cold shock genes, *csp*D1 and *csp*D2, was altered in NRC-1 cells during growth at 15°C, with the former up-regulated 2.3-fold and the latter up-regulated 3.1-fold. In addition, three heat shock genes, *dna*K, *grp*E and *hsp*5, were down-regulated from 1.5- to 4.8-fold in the cold. Most of these changes, some of which were among the highest fold-changes observed in our investigations, were reversed when cells were heat shocked (see below).

A striking observation was that gas vesicle gene expression and content were increased when *Halobacterium *sp. NRC-1 was grown in the cold. In particular, the rightward transcribed genes of the buoyant gas vesicle gene cluster (*gvp*ACNO coded on both pNRC replicons), were significantly up-regulated, showing increases from 1.8- to 8.0-fold in the cold. The first two genes of the leftward operon, *gvp*DE, which encode putative regulators for gas vesicle biosynthesis, were also up-regulated (3- and 3.4-fold). Microscopic examination of cells yielded results that were consistent with the microarray data, i.e. an increase in gas vesicle content observable in cells grown at lower temperatures (data not shown).

Previously, increases in the supercoiling of DNA have been reported upon cold shock in *E. coli *[45,46]; congruently, the levels of certain DNA topoisomerases change during cold shock [47,48]. Surprisingly, the *Halobacterium *sp. NRC-1 *gyr*B gene was found to be down-regulated 1.4-fold in the cold, and we did not observe significant changes in *gyr*A, *top*6AB, or *top*A. Interestingly, the archaeal histone gene (*hpy*A) was down-regulated (1.5-fold), while the actin (*mbl*) and tubulin-like (*fts*Z2) genes were up-regulated (1.7- and 1.6-fold respectively) in the cold, suggesting reduced need for genomic compaction but increased requirement for intracellular organization in the cold.

At reduced temperatures, the solubility of gases and the stability of toxic reactive oxygen species may increase [49,50]. However, we did not observe a corresponding increase in either superoxide dismutase (*sod*1 and *sod*2) gene or the glutathione S-transferase (*gst*) gene. Further, the peptidyl-prolyl *cis*-*trans *isomerase (*sly*D) and the peptidyl-prolyl isomerase (*ppi*A) genes were not up regulated. These results suggest that at low temperatures, NRC-1 does not enhance the use of these gene products to remove oxygen radicals or interact with hydrophobic patches and aid in the folding of proteins [51].

A variety of important genes necessary for metabolism and cellular functions were altered during growth at 15°C, likely as a result of decreased growth rate. Genes coding for the H^+^-transporting ATP synthase (*atp*IKEBD), several of the 30 and 50S ribosomal proteins (*rps *and *rpl *genes), transport (*sec*E, *gsp*E2), purine/pyrimidine metabolism (*pur*SQ, *apt*, *cmk*, *pyr*BI), and chemotaxis (*che*Y) were all down-regulated (1.4 to 3.0-fold). For genes of carbohydrate metabolism, *fba*A, a sugar aldolase, and *pmu*2, a sugar kinase, were about 1.7-fold down-regulated. Among genes related to peptide metabolism, e.g. the dipeptide transporter coded by the *dpp *gene, *dpp*A, was up-regulated (1.6-fold), as were two aminopeptidase genes, *pep*Q2 (3.4-fold) and *yux*L (2.1-fold). These findings suggest an effort to increase the amino acid pool available to cells in the cold; however, decreased growth rate would result from inhibition of ATP production, protein synthesis and export, DNA metabolism, and taxis.

### Heat shock

**Figure 7:**
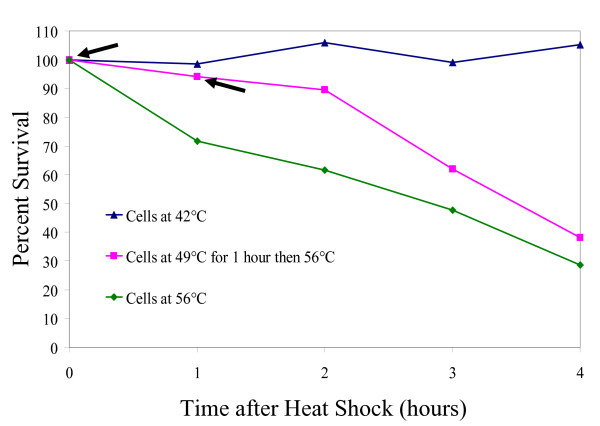
Percent survival of *Halobacterium *sp. NRC-1 after heat shock at 56°C with or without incubation at an intermediate temperature (49°C). Arrows indicate the point where cultures were harvested for microarray analysis.

*Halobacterium *sp. NRC-1 is known to be slightly thermotolerant, exhibiting relatively normal growth up to 48°C. As the temperature is increased from the growth optimum of 42°C, the growth rate declines and temperatures above 50°C prevent growth and provoke photobleaching. After treatment of cells at 49°C, normal growth resumes after shifting back to more moderate temperatures. Pretreatment of NRC-1 cells at 49°C for 1 hour, and then shifting to 56°C, increased survival 2.5-fold compared to cells directly shifted from 42 to 56°C, indicating a classic heat shock effect (Fig. 7) [52].

**Figure 8:**
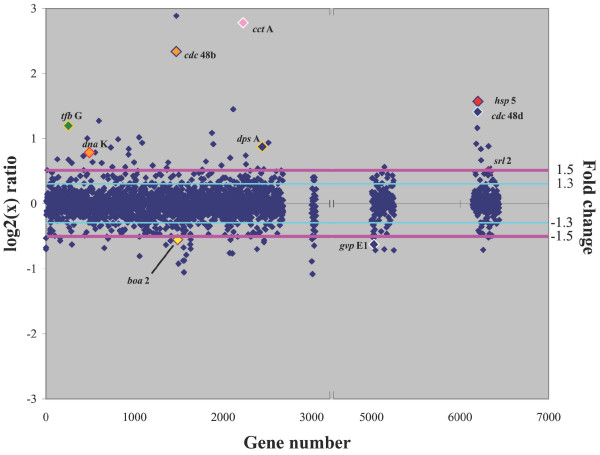
Scatter plot comparing DNA microarrays hybridized with cDNA from *Halobacterium *sp. NRC-1 cultures with or without heat shock (at 49°C). The log_2 _value of the *Cy*5/*Cy*3 ratio for each gene (abscissa) is plotted versus the gene number (ordinate). Gene numbers correspond to the chromosomal genes (1–2679), RNA genes (3000 to 3051), pNRC100 genes (5000 to 5256), and pNRC200 genes (6000 to 6487). Pink line represents a fold change value of 1.5 and the light blue line represents a fold change value of 1.3. Colors have been added for emphasis.

In order to determine which genes are responsive to heat shock, *Halobacterium *sp. NRC-1 cultures which were shifted for one hour from 42 to 49°C were compared to cultures remaining at 42°C. Heat shock at 49°C resulted in up-regulation of 64 genes and down-regulation of 43 genes by 1.5-fold or greater (Fig. 8). Genes of unknown function constituted 34% of the up-regulated and 28% of the down-regulated genes.

Of the four genes coding small heat shock proteins belonging to the Hsp20/α-crystallin family in *Halobacterium *sp. NRC-1, which are known to protect against irreversible aggregation of cellular proteins and assist in protein refolding during stress conditions [53], *hsp*5 was the most highly affected, being up-regulated 3.0-fold after heat shock (Table 4). Among genes encoding the *dna*K/*dna*J/*grp*E Hsp70 family chaperones involved in disaggregation and reactivation of proteins [54], only the *dna*K gene was found to be highly up-regulated, 1.8-fold after heat shock. The *cct*A gene, coding the chaperonin-containing t-complex polypeptide (Cct) thermosome family was up-regulated 8.7-fold [53]. This family has been hypothesized to be involved in general environmental stress [55-57]; however, in this transcriptomic study, we only observed an increase in transcript under heat shock in NRC-1. In addition, members of the AAA^+ ^ATPase class have been found to be up-regulated during heat shock and aid in the refolding of proteins [58,59]. Two members of this class of genes, *cdc*48b (5.6-fold) and *cdc*48d (3.4-fold), the latter of which is coded by pNRC200, were significantly up-regulated during heat shock.

**Table 4:** Selected genes significantly altered under heat shock

Gene ID	Gene name	Former name	COG	Predicted function	Fold change values	log_2_(x) ratio	Standard deviation of log_2_(x) ratio
254	tfbG		1405	transcription initiation factor IIB	2.412	1.199	0.450
491	dnaK		443	heat shock protein	1.747	0.786	0.233
1472	cdc48b		464	cell division cycle protein	5.631	2.340	0.637
1488	boa2		3413	bacterio-opsin activator-like protein	-1.471	-0.553	0.107
2226	cctA		459	thermosome subunit alpha	8.712	2.777	1.038
2443	dpsA		783	DNA protecting protein under starved conditions	1.854	0.876	0.205
5028	gvpE1			GvpE protein	-1.553	-0.626	0.162
6199	cdc48d		464	cell division cycle protein	3.371	1.413	1.080
6201	hsp5		71	heat shock protein	3.050	1.573	0.313
6320	srl2	vng6320		Smc and Rad50 like ATPase	1.471	0.493	0.420

Some researchers have reported an increase in DNA repair proteins during heat shock responses [60,61]. In our experiments, about 5% of the up-regulated genes were involved with DNA metabolism; however, only one DNA repair gene, *srl*2, a SMC/Rad50-like ATPase found on pNRC200 was slightly up-regulated (1.5-fold). Among other genes coding DNA binding proteins, we also observed an increase in *dps*A (1.9-fold), a stress inducible DNA binding protein, consistent with an earlier proteomic study [52].

Many genes seem to be coordinately regulated when *Halobacterium *sp. NRC-1 is exposed to the cold and heat, including twenty-five genes that were inversely regulated under the two conditions. Although many of these genes are of unknown function, several genes known to be involved in heat shock (*hsp*5, *dna*K, *cct*A), as well as two putative regulators (*gvp*E1 and *boa*2) were identified. In addition, ten genes were regulated in the same direction under both conditions, including *tfb*G (2.4-fold up-regulated), one of seven TFB genes in NRC-1.

## Conclusion

Using whole genome microarrays, we have identified genes likely to be involved in adaptation of the model halophilic Archaeon *Halobacterium *sp. NRC-1 to environmental stresses. Under all environmental stresses examined thus far, well-characterized stress response genes were observed; however, expression changes in unidentified or novel genes were also common. Heat shock showed induction of several chaperone genes, likely to protect cellular proteins from denaturation and breakdown. Growth in the cold suggested the alteration of lipid metabolism, an increased potential for flotation (to escape to a warmer zone) and a slowdown of protein production (possibly in preparation for dormancy). Growth in high salinity resulted in down-regulation of selected ion transporters, presumably to reduce the entry of toxic species. Responses to growth in low salinity also pointed to the need to maintain proper intracellular ionic conditions via the modulation of many transporter genes. Further bioinformatic and genetic analysis of the genes responsive to the many stressors that halophilic archaea respond to in their environment will lead to a fuller understanding of the biology of these interesting microbes.

## Methods

### Culturing and DNA microarray analysis

*Halobacterium *sp. NRC-1 cultures were grown in standard CM^+ ^medium with shaking at 220 rpm on a New Brunswick Scientific platform shaker. For growth in the cold, cultures were aerobically grown at 15°C. For heat shock experiments, cells were grown at 42°C followed by incubation at 49°C for one hour and 56°C for three or four hours. For salt experiments, cells were grown at 42°C with either 2.9, 4.3, or 5.0 M NaCl. Pre-cultures grown under the same conditions used in the growth curve were used as inocula. For the heat killing profile, *Halobacterium *sp. NRC-1 cultures used were grown under standard laboratory conditions (aerobically at 42°C in CM^+ ^medium). Each growth curve and heat killing profile was based on the average of three cultures. For each microarray experiment, three cultures were grown in the same manner as those used for the growth curves and RNA was pooled from all three cultures for cDNA synthesis and hybridization. Cultures grown at high and low salinities and 15°C were harvested at early exponential phase (OD_600 _= 0.19 to 0.23) for microarray analysis. Cultures used for the heat shock microarrays were grown to early exponential phase and then incubated at 49°C for 1 hour. Before harvesting the cells to collect RNA, cultures were swirled briefly in an ethanol-dry ice bath to rapidly cool the cultures and "freeze" the RNA profile. Total RNA was isolated using the Agilent total RNA isolation mini kit (Agilent Technologies, Palo Alto, CA) and then treated with RNase-free DNase I. cDNA was prepared with equal amounts of RNA from control and experimental samples and then fluorescently labeled with *Cy*3-dCTP and *Cy*5-dCTP. Concentrations of RNA and cDNA were measured using a Nanodrop (ND-1000) spectrophotometer (NanoDrop Technologies, Wilmington, DE). Incorporation of the *Cy*3 and *Cy*5 labels was checked via gel electrophoresis and scanning on a GE Typhoon fluorescence scanner (GE Healthcare, Piscataway, NJ). Washing and hybridization of the arrays was performed as recommended by Agilent and as previously described [5]. Slides were scanned for *Cy*-3 and *Cy*-5 signals with an Agilent DNA-microarray scanner. Probe signals were extracted with the Agilent Feature Extraction Software and analyzed using the statistical methods described below. Oligonucleotide arrays, *in situ *synthesized using ink-jet technology, were used for transcriptome analysis of *Halobacterium *sp. NRC-1. Oligomer (60-mer) probes were previously designed for 2474 ORFs utilizing the program OligoPicker [16]. Two replicate microarrays were performed for the high and low salinity and 15°C growth experiments, and three replicate microarrays were performed for the heat shock experiment. Data shown are based on the analysis of all arrays performed for each of the given conditions.

### Microarray data processing

The Agilent Feature Extraction program was used for image analysis and processing of the microarray image file. Background signals were subtracted from raw signals using the area either on or around the features. Dye biases created by differences in the red and green channel signals caused by different efficiencies in labeling, emission and detection were also estimated and corrected. Signals from each channel were normalized using the LOWESS algorithm to remove intensity-dependent effects within the calculated values. Further statistical analyses were performed as described below.

### Statistical analysis

The illuminant intensity, log_2_(x) value, and standard deviation of the log_2_(x) value were calculated for the normalized red and green probe values for each gene in each microarray. The illuminant intensity was calculated through the logarithm of the geometric mean of *Cy*5 and *Cy*3 processed signal intensities by first calculating:$probesignal=\frac{1}{2}\log_{2}(Cy5*Cy3)$. Then for a finite-sized sample of size N, we calculated the intensity using the arithmetic mean: $\text{illuminant intensity}=1/\text{N}\sum_{i=1}^{n}probesignal_{i}$. Biased estimators for sample means of log_2_(x) ratios were calculated by the arithmetic mean of log_2_(x) ratios for a set of N probes for a gene; where *r*_*i *_is the processed *Cy*5 illuminant intensity level and *g*_*i *_is the processed *Cy*3 illuminate intensity value for the *i*th probe: $\bar{\log_{2}}=1/\text{N}\sum_{i=1}^{n}\log_{2}(r_{i},g_{i})$[62]. Standard deviations for sample means of log_2_(x) ratios were then calculated: ${\left(1/N\sum_{i=1}^{n}{\left(\log_{2}(r_{i,}g_{i})-\bar{\log_{2}}\right)}^{2}\right)}^{1/2}$. Changes in transcript levels were considered significant if they were changed more than 1.3- to 1.5-fold using a linear transform function, with the upper figure used to calculate total numbers of altered genes under specific conditions. See Tables 1, 2, 3, 4 for a listing of notable regulated genes, their fold change, log_2_(x) value, and standard deviation under the high and low salinity and temperature conditions tested.

### Database and cluster analysis

All microarray data were stored in our HaloArray database [63] mirrored on two Linux servers. The systems use Apache server software, MySQL databases, and custom Perl script-based webtools designed for access and data analysis. This database is a part of our comprehensive HaloWeb database [64], which includes our genome sequence and annotation data, and is indexed in the Thompson ISI Web of Knowledge and Current Web Contents.

## Competing interests

The author(s) declare that they have no competing interests.

## Authors' contributions

SD conceived the study, and JAC and PD conducted the experimental analysis, and together with SD, analyzed the data and wrote the manuscript. JAC and JK did the statistical analysis and JK developed the HaloArray database and microarray analysis tool, and JAM designed the microarrays and initiated the DNA microarrays experiments. All authors read and approved the final manuscript.
